# Imaging of excised cochleae by micro-CT: staining, liquid embedding, and image modalities

**DOI:** 10.1117/1.JMI.10.5.053501

**Published:** 2023-09-25

**Authors:** Jannis Justus Schaeper, Michael Charles Liberman, Tim Salditt

**Affiliations:** a University of Göttingen, Institute for X-ray Physics, Göttingen, Germany; b University of Göttingen, Cluster of Excellence “Multiscale Bioimaging: Molecular Machines to Networks of Excitable Cells,” Göttingen, Germany; c Massachusetts Eye and Ear Infirmary, Eaton-Peabody Laboratories, Boston, Massachusetts, United States; d Harvard Medical School, Department of Otolaryngology, Head and Neck Surgery, Boston, Massachusetts, United States

**Keywords:** three-dimensional histology of the cochlea, x-ray phase-contrast tomography, propagation-based phase contrast, x-ray computed-tomography, heavy-metal staining, synchrotron radiation, compact μ-CT, multi-scale imaging

## Abstract

**Purpose:**

Assessing the complex three-dimensional (3D) structure of the cochlea is crucial to understanding the fundamental aspects of signal transduction in the inner ear and is a prerequisite for the development of novel cochlear implants. X-ray phase-contrast computed tomography offers destruction-free 3D imaging with little sample preparation, thus preserving the delicate structure of the cochlea. The use of heavy metal stains enables higher contrast and resolution and facilitates segmentation of the cochlea.

**Approach:**

For μ-CT of small animal and human cochlea, we explore the heavy metal osmium tetroxide (OTO) as a radiocontrast agent and delineate laboratory μ-CT from synchrotron CT. We investigate how phase retrieval can be used to improve the image quality of the reconstructions, both for stained and unstained specimens.

**Results:**

Image contrast for soft tissue in an aqueous solution is insufficient under the in-house conditions, whereas the OTO stain increases contrast for lipid-rich tissue components, such as the myelin sheaths in nervous tissue, enabling contrast-based rendering of the different components of the auditory nervous system. The overall morphology of the cochlea with the three scalae and membranes is very well represented. Further, the image quality of the reconstructions improves significantly when a phase retrieval scheme is used, which is also suitable for non-ideal laboratory μ-CT settings. With highly brilliant synchrotron radiation (SR), we achieve high contrast for unstained whole cochleae at the cellular level.

**Conclusions:**

The OTO stain is suitable for 3D imaging of small animal and human cochlea with laboratory μ-CT, and relevant pathologies, such as a loss of sensory cells and neurons, can be visualized. With SR and optimized phase retrieval, the cellular level can be reached even for unstained samples in aqueous solution, as demonstrated by the high visibility of single hair cells and spiral ganglion neurons.

## 1 Introduction

The cochlea is the receptor organ in the inner ear that transduces sound into neuronal activity. Fundamental aspects of signal transduction and neuro-physiology as well as biomedical research (implant technology,^1^ hearing loss, and disorders^2^), all call for three-dimensional (3D) imaging techniques to quantify the micro-anatomy and histology, from organ down to the cellular and sub-cellular levels. In view of the organ’s intricate and subtle structure, non-destructive and structure-preserving approaches are desirable for *post mortem* imaging of excised human cochlea. This also applies to small animal models, which are indispensable in the development of novel implant technology or treatments. With respect to 3D imaging, conventional histology faces several major deficits and restrictions. Apart from possible slicing or staining artifacts, it is tedious and time-consuming to record the entire organ or large field of views (FOVs) by parallel sections, making it almost impossible to cover the complete 3D cyto-architecture of a specimen, even at moderate resolution. By contrast, x-ray phase-contrast computed tomography (XPCT)^3^ is able to assess the native 3D structure of tissues with selectable FOV, including the micro-anatomical and the histological range, such as with FOVs of several centimeters covering the entire organ or several millimeters covering a region of interest (ROI). Voxel sizes can be chosen accordingly, in the range of a few microns (μ-CT) or even in the sub-μm (nano-CT) range, when high-resolution detectors and/or magnification in cone-beam geometries is used. Numerous recent μ-CT imaging studies of cochlea have spurred interest in this imaging modality for various applications, from creating basic digital twins for modeling^4^ to the investigation of specific pathologies^5^ and image-guided implant positioning^6^ and quality control.^7^

The advantages of synchrotron radiation (SR) phase contrast for radiography of cochlear tissue have been demonstrated, both for in-line phase contrast^8^ and for grating interferometry.^9^ X-ray absorption contrast studies include buffered small-animal cochleae^10^ and human cochlea with metal implants.^11^ The usefulness of in-house μ-CT for medium resolution overview scans of dried small-animal cochleae has been demonstrated, using phase contrast even at sources of low brilliance/coherence.^12^ Phase-contrast μ-CT of dried cochlea was also reported with SR at a compact source,^13^ with very satisfactory image quality overall. However, structure preservation in dried specimens is severely compromised and, hence, of limited value. Using XPCT with phase sensitivity arising from free-space propagation between the sample and detector in combination with highly brilliant undulator radiation, we finally achieved excellent image quality in cochleae that were not dried but kept in alcohol/solvent.^14^ Importantly, we also reached the cellular level required for (3D) histology and histopathology,^14^ targeting the spiral ganglion and the basilar membrane of intact small animal cochleae. The specimens were scanned in ROI (local) tomography mode, while immersed in methanol or dibenzylether, for uncleared and cleared cochleae, respectively. Tissue clearing was applied to enable correlative imaging by μ-CT and light sheet microscopy.^14^ Hydrated cochleae in phosphate buffer (PBS), however, did not yield sufficient contrast in unstained cochleae. XPCT with SR has also been used for human cochlea,^15^ including tonotopic mapping of the human cochlea at 9  μm voxel size.^16^ μ-CT versus SR phase-contrast imaging of human and mammalian cochlea was also previously compared^17^ but not down to the histological level.

In this work, we explore heavy metal stains that are well known from electron microscopy as radiocontrast agents for μ-CT of cochleae, including XPCT with laboratory μ-CT instruments and SR. Note that radiocontrast agents have been used in a number of CT and XPCT studies.^18^^,^^19^ Contrast enhancement by heavy metals, in particular fixation or post-fixation with osmium tetroxide (OTO, ${\mathrm{OsO}}_{4}$), has also been used for cochlear CT in absorption contrast^20^^–^^22^ as it provides superior contrast for nerve fibers and membranous structures. This has been exploited to investigate malformations^23^ and in relation to conventional histology.^24^^,^^25^ The benefit of staining not only for absorption but also for XPCT has been demonstrated convincingly and was used for cochlear synaptopathy in Ref. 5. The present work now reports further advances in high-resolution XPCT of cochleae (including unstained cochleae), using both laboratory μ-CT as well as high-brilliance SR based on contrast formation by free-space propagation, offering higher resolution than grating or crystal-based XPCT.^26^ Note that XPCT at in-house μ-CT instruments is challenging because phase-sensitive image formation by propagation requires at least partial coherence, which is now available even at compact in-house μ-CT sources due to smaller spot sizes and higher brilliance.^12^^,^^13^^,^^27^^–^^29^ For much better established XPCT with SR, on the other hand, optimization of image acquisition and phase retrieval parameters can still be challenging,^30^^,^^31^ in particular for heavy metal stains. This is because stained samples can easily violate the common weak object assumption in phase retrieval, on which the contrast-transfer function (CTF) method is based.^32^ Therefore, XPCT of metal-stained samples has to be carried out at a suitable photon energy E, which has to be increased with respect to the energy range E≃7…14  keV, used in our earlier SR-studies of unstained small animal cochlea, with cross section up to a few millimeters. As we show here, increasing the energy to the range E≃16…21  keV gives satisfactory results also for heavy metal stained samples scanned with SR. For laboratory instruments, a broad spectrum is exploited, and it can be adjusted and tailored to the sample by choosing a suitable peak voltage and eventually pre-hardening based on filters.

After this introduction, the methods concerning phase retrieval algorithms, setup image acquisition, rendering, and sample preparation are given, followed by the results for laboratory μ-CT and SR. The manuscript closes with a brief discussion and outlook section.

## 2 Methods

### 2.1 Phase Retrieval

We employ propagation-based phase contrast. The imaging regime is determined by the unitless Fresnel number $F={\mathrm{px}}_{\mathrm{eff}}^{2}/z_{\mathrm{eff}}\lambda$, where λ denotes the wavelength, ${\mathrm{px}}_{\mathrm{eff}}=\mathrm{px}/M$ is the effective pixel size, and $z_{\mathrm{eff}}=z_{12}/M$ is the effective propagation distance. Phase contrast is governed by the real-part decrement $\delta (\vec{r},E)$ of the x-ray refractive index $n(\vec{r},E)=1-\delta (\vec{r},E)+i\beta (\vec{r},E)$. For the data recorded at the μ-CT setup with low coherence at F≃1 in the direct contrast regime, the phase contrast manifests itself in the form of edge enhancement. A suitable reconstruction that preserves image sharpness is given by the so-called scheme of Bronnikov-aided correction (BAC).^33^^,^^34^ It is based on the transport of intensity equation (TIE), which relates the projected object phase $\phi (\vec{r})$ to the derivative of the intensity along the optical axis $\partial_{z}I(\vec{r})$:

$$\nabla_{⊥}(I(\vec{r})\nabla_{⊥}\phi (\vec{r}))=-k\partial_{z}I(\vec{r}),$$ (1)

where the wave number k=2π/λ. Under the assumptions of a weakly absorbing object and small propagation distances, the TIE is linearized and solved for the phase, which is the essence of the so-called modified Bronnikov algorithm:^35^^,^^36^

$$\overset{˜}{\phi }({\overset{\to }{r}}_{⊥})=2\pi F\cdot F_{⊥}^{-1}[\frac{F_{⊥}[I({\overset{\to }{r}}_{⊥},z)/I_{0}-1]}{{\left|{\overset{\to }{k}}_{⊥}\right|}^{2}+\alpha }],$$ (2)

where F is the Fourier transform, $I_{0}$ is the intensity of the illumination, and α is a regularization parameter. The approximated phase $\tilde{\phi }$ is used to obtain a sharp intensity distribution behind the object^33^ according to

$$I({\overset{\to }{r}}_{⊥},z=0)=\frac{I({\overset{\to }{r}}_{⊥},z)}{1-\gamma \nabla_{⊥}^{2}\overset{˜}{\phi }({\overset{\to }{r}}_{⊥})}.$$ (3)

The SR-XPCT data were recorded in a parallel beam geometry at defocus distances in the holographic regime at a Fresnel number F=0.03. Due to the high coherence, phase retrieval was based on the CTF, which is suitable for homogenous, weakly absorbing objects (β/δ=const.):^32^^,^^37^

$$\frac{I({\overset{\to }{k}}_{⊥},z)}{I_{0}}=2\pi \delta_{D}({\overset{\to }{k}}_{⊥})+2\phi ({\overset{\to }{k}}_{⊥})\sin(\chi )-2\mu ({\overset{\to }{k}}_{⊥})\cos(\chi ),$$ (4)

where $\chi =\frac{\lambda zk_{x}^{2}}{4\pi }$, the Dirac-delta function is $\delta_{D}({\vec{k}}_{⊥})$, and the absorption coefficient is $\xi ({\vec{k}}_{⊥})$. The phase behind the object is computed from a single-step calculation,^37^ applying a regularized Fourier filter adapted to the CTF:

$$\phi ({\overset{\to }{r}}_{⊥})=F_{⊥}^{-1}[\frac{\left(\sin(\chi_{d})+\beta /\delta \;\cos(\chi_{d}))\cdot F_{⊥}[I({\overset{\to }{r}}_{⊥},z_{d})-1\right]}{2(\sin(\chi_{d})+\beta /\delta \;\cos(\chi_{d}){)}^{2}+\alpha ({\overset{\to }{k}}_{⊥})}],$$ (5)

where the frequency-dependent regularization parameter is $\alpha ({\vec{k}}_{⊥})$. The regularization parameters for phase retrieval were chosen manually by visual inspection. All phase retrieval functions were used as implemented in the published HoloTomoToolbox.^31^

### 2.2 Experimental Setup

#### 2.2.1 Laboratory μ-CT setup

Tomographic scans were acquired using a μ-CT instrument (EasyTOM, RX Solutions) equipped with a microfocus (Hamamatsu L12161-07, W Target, 5 to 50  μm spotsize) and a nanofocus source (Hamamatsu L10711-02, W target, ${\mathrm{LaB}}_{6}$ cathode). Projection images were recorded by a CCD camera (4008×2672  pixels, 9  μm pixel size) equipped with a fiber coupled Gadox scintillator, or alternatively by a flat panel detector (1440×1704  pixels, 127  μm pixel size). For all samples, an overview scan and an ROI scan were performed. The projections of the ROI scans were phase-reconstructed with the BAC-algorithm as implemented in the holoTomoToolbox.^31^ The tomographic cone beam reconstruction was performed with the software of the instrument. The resolution was determined with a JIMA absorption target, consisting of lines and spaces of different spacings. The acquisition parameters and the parameters of the reconstruction volume for the data presented here are tabulated in Table 1.

**Table 1:** Acquistion parameters for the tomographic scans recorded with the laboratory μ-CT setup.

Parameter	Overview guinea pig	ROI guinea pig	Overview human	ROI human
Source	Nanofocus	Nanofocus	Microfocus	Nanofocus
Source spot mode	Large focal spot	Small focal spot	Small focal spot	Large focal spot
Energy E (kV)	80	60	80	80
Detector	CCD	CCD	Flat panel	CCD
Counting time (s)	8 × 4	20 × 5	99 × 0.36	20 × 6
Projections	1568	1568	1120	1568
$z_{01}$	38.4 mm	11.2 mm	35.4	21.2
$z_{02}$	138.4 mm	203.4 mm	623.0	128.4
FOV (${\mathrm{mm}}^{2}$)	6.3 × 10.0	2.0 × 1.3	10.4 × 12.3	6.0 × 3.7
Voxel size $dx_{\mathrm{eff}}$ (μm)	5.0	1.0	7.2	3.0
Resolution (JIMA) (μm)	7	5	10	7
Fresnel number F	—	1.5202	—	6.0839
BCA α	—	0.1	—	0.2
BAC γ	—	0.9	—	0.8

#### 2.2.2 Synchrotron setup

**Fig. 1:**
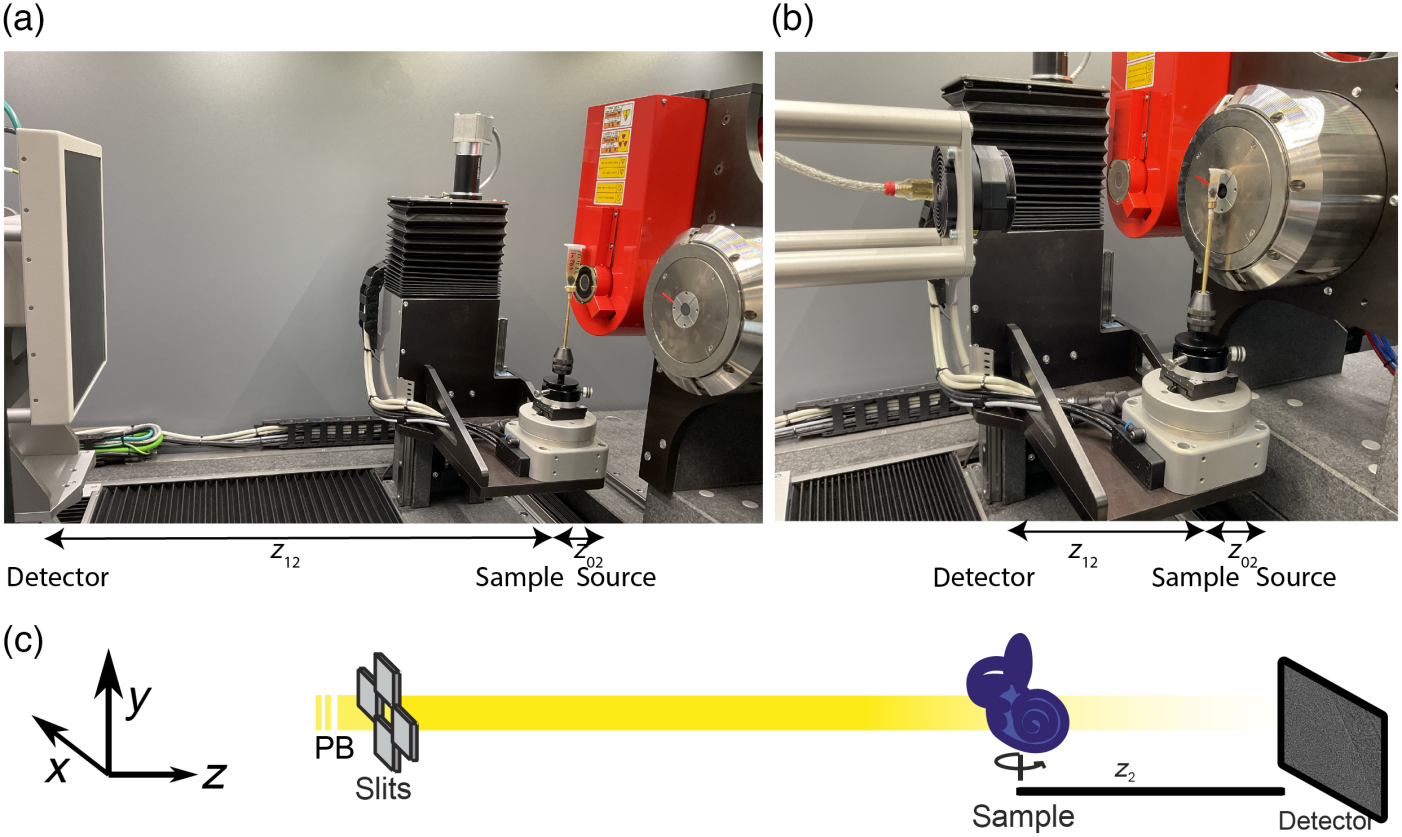
Experimental setups. Laboratory μ-CT instrument (EasyTOM, RX Solutions) with (a) microfocus source and flat panel detector and (b) nanofocus source and CCD detector. The system can be operated in all source–detector combinations. (c) Synchrotron parallel beam setup at the GINIX endstation of the P10 coherence beamline (PETRA III, DESY).

High-resolution scans were performed at the GINIX nanofocus endstation^38^ of the P10 coherence beamline at the PETRA III storage ring (DESY, Hamburg) in a parallel beam geometry.^30^ The setup is sketched in Fig. 1(c). The undulator radiation is monochromatized by a Si-111 double crystal monochromator to a photon energy E=21  keV to ensure a large penetration depth. Images were acquired with a pco edge 5.5 sCMOS camera mounted on an Optique Peter system with a 50  μm LuAG:Ce scintillator and a 10-fold objective. In a parallel beam geometry, this results in an effective pixelsize of 650 nm. Due to high-beam intensity, the SR beam was attenuated by 1.6 mm (64×25  μm) Si foils. A continuous rotation of the sample stage and fast tomographic data acquisition with a counting time of 35 ms were used, resulting in scan times of 2 min per tomogram. Tomograms were acquired at a single distance with $z_{12}=29\;\mathrm{mm}$, corresponding to the direct contrast regime with F=0.0326. Phase reconstruction was performed by CTF [Eq. (5)] as implemented in the holoTomoToolbox.^31^ Tomographic reconstruction was performed with the filtered backprojection implementation of the astra toolbox.^39^^,^^40^ For an upper bound of the resolution, the Fourier shell correlation (FSC) was calculated. The acquisition parameters and the parameters for the reconstruction of the synchrotron dataset are tabulated in Table 2.

**Table 2:** Acquisition parameters for synchrotron phase-contrast tomography.

Parameter	PB
Energy E	21 keV
Detector	pco (10-fold objective)
Counting time	35 ms
Projections	3000
Scan range (deg)	0 to 360
Distances	1
$z_{01}$	88 m
$z_{02}$	29 mm
FOV (${\mathrm{mm}}^{2}$)	1.6 × 1.4
Voxel size $dx_{\mathrm{eff}}$	650 nm
Resolution (FSC)	3.5 μm
Fresnel number F	0.0326
Imaging regime	Direct contrast
CTF β/δ	1/45
CTF $\alpha_{1}$	$10^{-4}$
CTF $\alpha_{2}$	$10^{-1}$

### 2.3 Volume Renderings

The virtual slices and maximum intensity projections (MIPs) were created with Avizo (Thermo Fischer Scientific, Waltham, California, United States). The reconstructions were rendered using the 3D volumetric visualization framework NVIDIA Index (NVIDIA Corporation, Santa Clara, California, United States).

### 2.4 Sample Preparation

Guinea pigs were used to investigate noise-induced damage to the cochlea.^41^ The animals were exposed to an intense noise band at 4 to 8 kHz at 116 dB SPL for 2 h. This type of exposure is expected to produce damage to the sensory cells and their associated nerve fibers, localized to the cochlear region tuned to frequencies near the peak of the noise spectrum.^42^ At 2 months post-exposure, the cochleae were fixed by intravascular perfusion with paraformaldehyde, removed from the skull and post-fixed in the same solution overnight. After fixation, some cochleae were stained with OTO (${\mathrm{OsO}}_{4}$) for 1 h and imaged in PBS. The non-stained cochleae were dehydrated in an increasing methanol-series for better contrast. All procedures for animal anesthetization, acoustic overexposure, and cochlear extraction were approved by the Institutional Animal Care and Use Committee of the Massachusetts Eye and Ear Infirmary.

The human cochlea was removed at autopsy, immersion fixed in 4% formalin for several days, and then stained with OTO for 1 h. The appropriate pre-mortem human consent forms and all subsequent procedures for technical processing and HIPAA-compliant data storage for the temporal-bone study were approved by the Human Studies Committee of the Massachusetts Eye and Ear Infirmary.

All cochleae were gently fixed in Eppendorf tubes and immersed in the aqueous solution (osmicated samples) or in methanol (non-osmicated samples). OTO binds to lipids and is a staining method widely used in electron microscopy.^43^

## 3 Results

### 3.1 Lab μ-CT of the Guinea Pig Cochlea

**Fig. 2:**
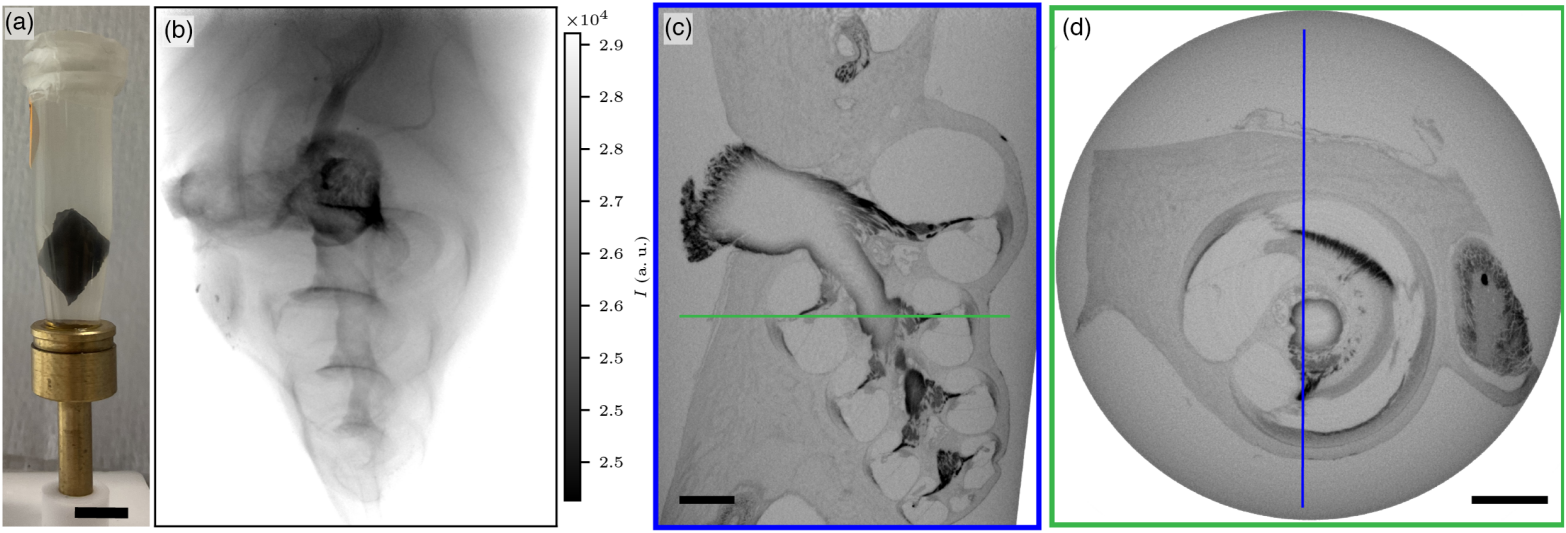
μ-CT overview scan of OTO-stained guinea pig cochlea acquired with the nanofocus source and CCD camera at a voxel size of 5  μm. (a) Photo of the sample mounted for x-ray tomography, scale bar 0.5 cm. (b) Example projection image. (c) Virtual reconstructed xz-slice, scale bar 1 mm. (d) Virtual reconstructed xy-slice, scale bar 1 mm. The orientation of the slices in (c) and (d) is indicated with a blue and green rectangle, respectively. The dataset reveals the morphology of the cochlea with the three scalae and the central nerve trunk in the modiolus.

The first example selected here to illustrate the capabilities of the μ-CT setup is an OTO-stained guinea pig cochlea in PBS. Figure 2 shows the sample mounting, an example projection image, and virtual reconstructed slices, reconstructed at a voxel size of 5  μm from a scan with 1568 projection angles. Each projection was averaged over 8 images with a counting time of 4 s. The so-called nanofocus source (with sub-μm source size), which is an open transmission type source (see Sec. 2), was chosen, and images were recorded with the CCD camera. Although image contrast for soft tissue in an aqueous solution is insufficient under the in-house conditions, the OTO stain increases contrast for lipid-rich tissue components, such as the myelin sheaths in nervous tissue. Overall, the morphology of the cochlea with the three scalae and membranes is clearly represented.

### 3.2 Phase Retrieval for the μ-CT Dataset

**Fig. 3:**
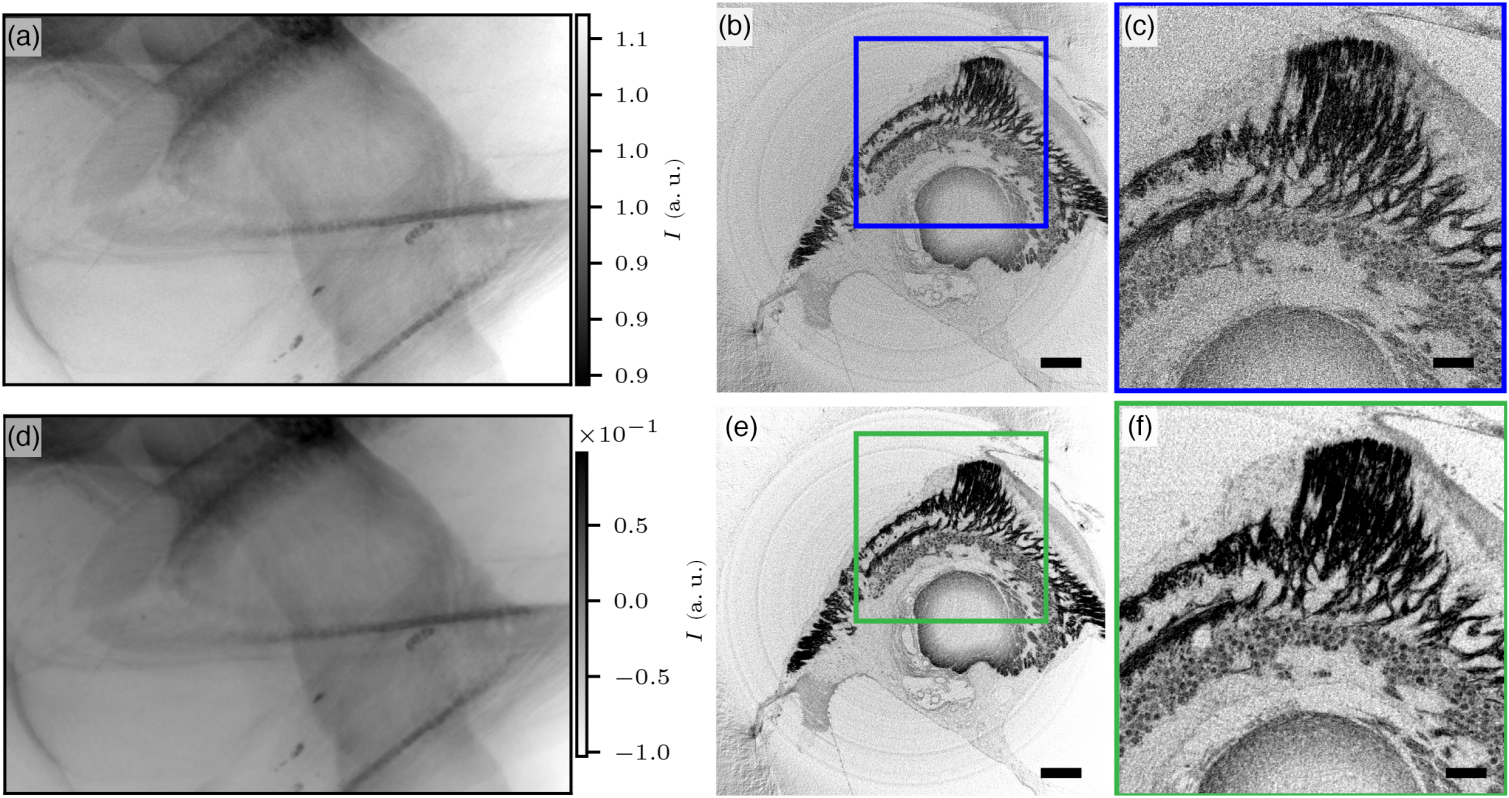
Phase retrieval at a μ-CT ROI scan of OTO-stained guinea pig cochlea with the BAC algorithm acquired with the nanofocus source and CCD camera at a voxel size of 1  μm. (a) Empty-beam corrected projection image, (b) virtual reconstructed xy-slice, scale bar 200  μm. (c) Zoom into (b), scale bar 100  μm. (d) BAC phase-reconstructed projection with α=0.1 and γ=0.9. (e) Reconstructed virtual xy-slice of (d), scale bar 200  μm. (f) Zoom into (e), scale bar 100  μm. Phase retrieval notably enhances image contrast.

Next, an ROI scan of the sample presented in Fig. 2 was recorded at a voxel size of 1  μm. This corresponds to a Fresnel number F=1.5202 in the direct contrast regime at which the phase contrast manifests itself as edge enhancement. In contrast to the overview scan, it makes sense at these parameters to test phase retrieval corrections. Figure 3 illustrates the ROI scan and an example slice, reconstructed with and without prior phase retrieval by the BAC scheme. In Fig. 3(a), an example projection is shown along with Figs. 3(b) and 3(c) slices through the reconstructed volume without prior corrections of the projections. By contrast, Fig. 3(d) shows the projection after phase retrieval by BAC, and Figs. 3(e) and 3(f) show the corresponding reconstructions computed after treating the projections by the BAC scheme. Although the projection image itself looks good without phase retrieval, the resulting edge-artifacts diminish the quality of the reconstructed 3D volume. This can be seen by comparing the reconstructed slices, in which the neurons and nerve fibers show higher contrast in the phase reconstructed dataset [see Figs. 3(e) and 3(f) compared with Figs. 3(b) and 3(c), respectively].

### 3.3 Imaging Unstained Guinea Pig Cochlea with Synchrotron Radiation

As we saw above, osmicated cochlea can be imaged very well with in-house μ-CT instrumentation, and phase retrieval improves the image quality for high-resolution scans. Next, we show that staining is not a prerequisite for liquid-embedded cochlear imaging when high-brilliance SR is used. To this end, we present results for an unstained guinea pig cochlea, scanned with a monochromatic (Si(111) monochromator) parallel beam at 21 keV photon energy. The solvent was changed to methanol to enhance the contrast between the solvent and the cochlear soft tissue. After flatfield correction, phase retrieval was performed by the CTF [Eq. (5)] with parameters β/δ=1/45, $\alpha_{1}=10^{-4}$, and $\alpha_{2}=10^{-1}$.

**Fig. 4:**
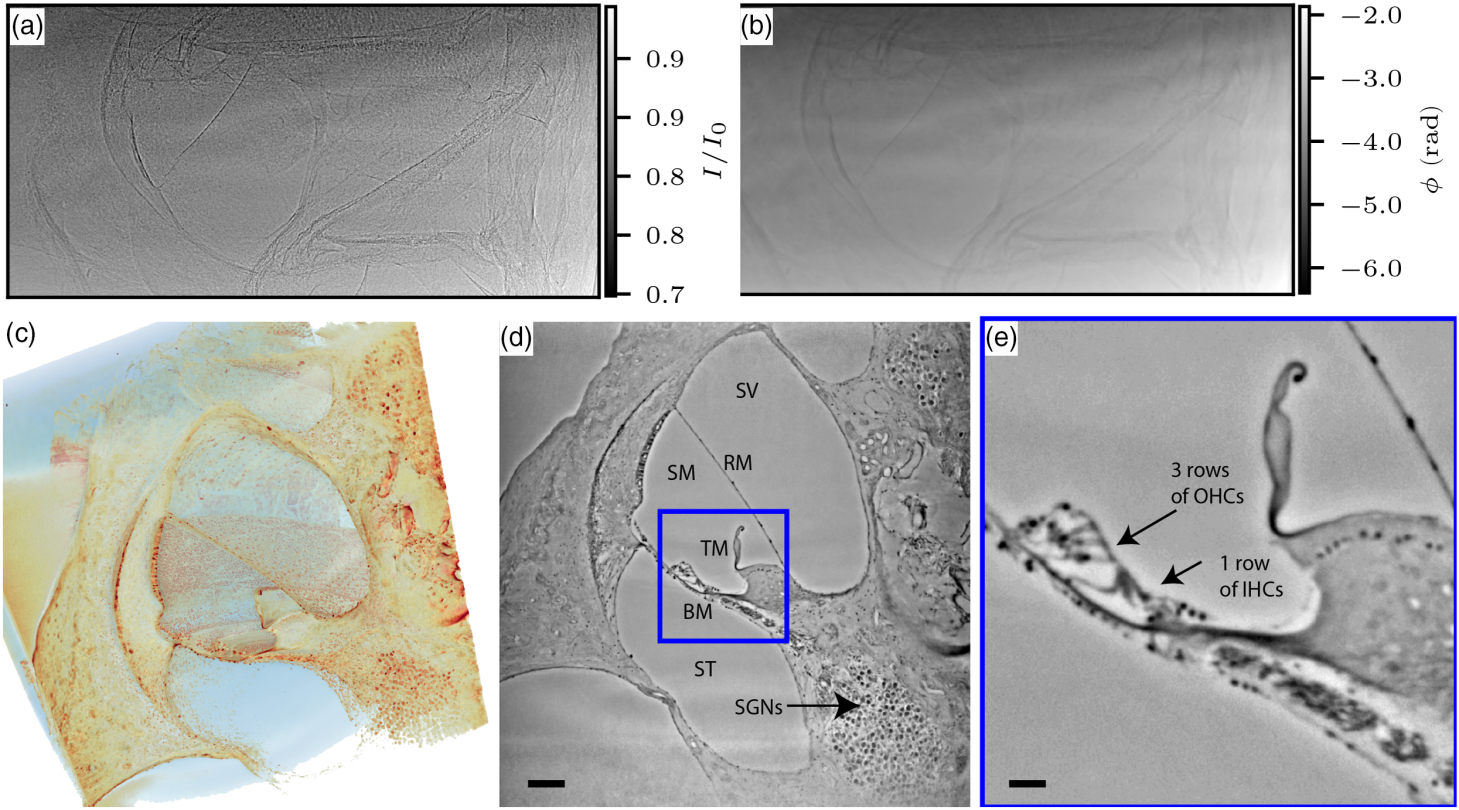
Synchrotron phase-contrast tomography of the unstained guinea pig cochlea in methanol. The tomograms were acquired at the GINIX endstation of the coherence beamline P10, PETRA III, DESY at an energy E=21  keV and with an effective pixelsize ${\mathrm{px}}_{\mathrm{eff}}=650\;\mathrm{nm}$. (a) Example projection. (b) CTF phase-reconstructed projection, β/δ=1/45, $\alpha_{1}=10^{-4}$, and $\alpha_{2}=10^{-1}$. (c) Volume rendering with NVIDIA IndeX. (d) Virtual reconstructed xy-slice, with the three scalae (ST, scala tympani; SM, scala media; and SV, scala vestibuli), separating membranes (RM, Reissner’s membrane; TM, tectorial membrane; and BM, basilar membrane), and spiral ganglion neurons, scale bar 100  μm. (e) Zoom into the organ of Corti with three rows of inner hair cells and one row of outer hair cells. scale bar 25  μm.

Figure 4 shows the results: starting with (a) a flat-field corrected projection and (b) the same projection after phase retrieval by CTF, as well as (c)–(e) slice renderings of the reconstructed volume. The flat-field correction can be challenging as slight movements (drifts and vibrations) of the monochromator result in artifacts, which are subsequently difficult to correct. Again the phase contrast effectively acts as a high pass, and fine tissue features are well represented in the untreated projection [see Fig. 4(a)], whereas phase retrieval with the regularized CTF scheme does not necessarily result in a more appealing projection [Fig. 4(b)] but is an indispensible and crucial step for the SR data. Figures 4(c)–4(e) demonstrate that 3D virtual histology can be achieved by XPCT of cochlea as single cells and cytoarchitecture are well represented. Note that the FOV is about 1.5 mm (depending on cropping of edges) in this setting, but the entire organ can be scanned by stitching of tomograms. The data of such a whole organ 3D map have been recorded without any sign of radiation damage.

### 3.4 Histopatholoy in Guinea Pig Datasets

Next we show how the in-house imaging can be applied to study cochlear pathology. To this end, the datasets of Figs. 2 and 3 were analyzed further. Because the cochlea originated from an animal exposed to intense noise, it is expected to produce a punctuate cochlear lesion, which can be further examined in 3D by XPCT.

**Fig. 5:**
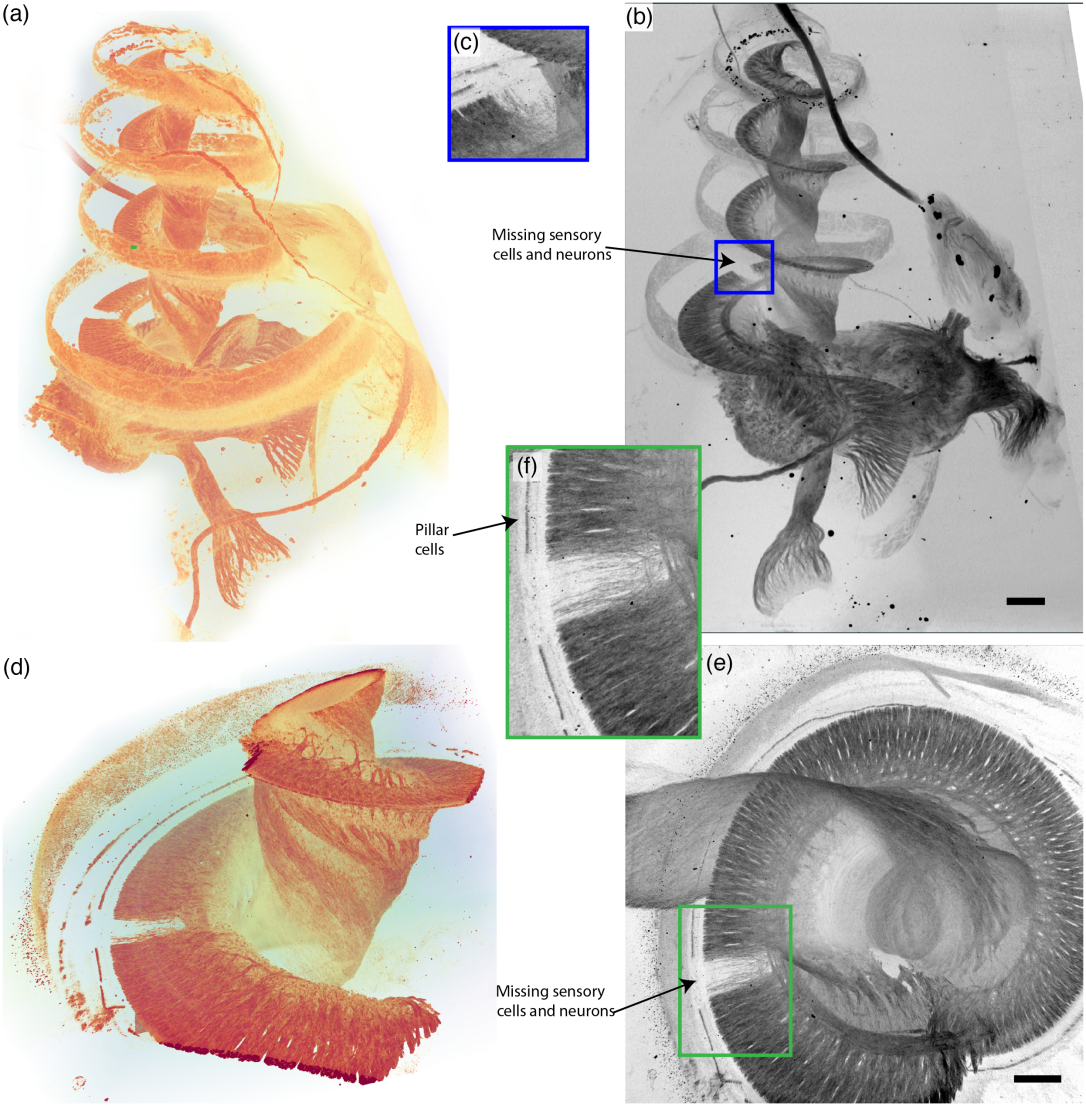
Visualizing the noise induced pathology of the overview and ROI scans of the guinea pig cochlea. In a specific area of the sensory epithelium that is normally tuned to the frequencies near the peak of the traumatic noise spectrum, the neurons and pillar cells are degenerated. (a) Volume rendering with NVIDIA IndeX. (b) MIP in the yz-direction, scale bar 400  μm. (c) Zoom into (b) as indicated by blue rectangle. (d) Volume rendering with NVIDIA IndeX. (e) MIP of ROI tomo in the xy-direction, scale bar 100  μm. (f) Zoom into (e).

Figure 5 shows volume renderings and MIPs, which help visualize the affected regions in 3D. As discussed above, the axons of cochlear nerve fibers are intensely stained by the OTO by virtue of their myelin sheaths. Within the sensory epithelium, the pillar cells are most intensely stained due to the highly organized microtubular bundles that they contain. The missing pillar cells and neurons at a specific location along the tonotopic axis corresponding to the peak of the noise spectrum can clearly be made out. The pathology is easily visualized already by an MIP because the sample only has high contrast (high absorption) where the OTO stain binds, and hence the nerve trunk and especially the spiralling peripheral axons of the cochlear nerve stand out in the MIP as they radiate from the nerve core to the spiraling sensory epithelium where they contact the hair cells. In this way, the spiral extent of the neural damage is easily visualized.

### 3.5 Lab μ-CT of Human Cochlea

**Fig. 6:**
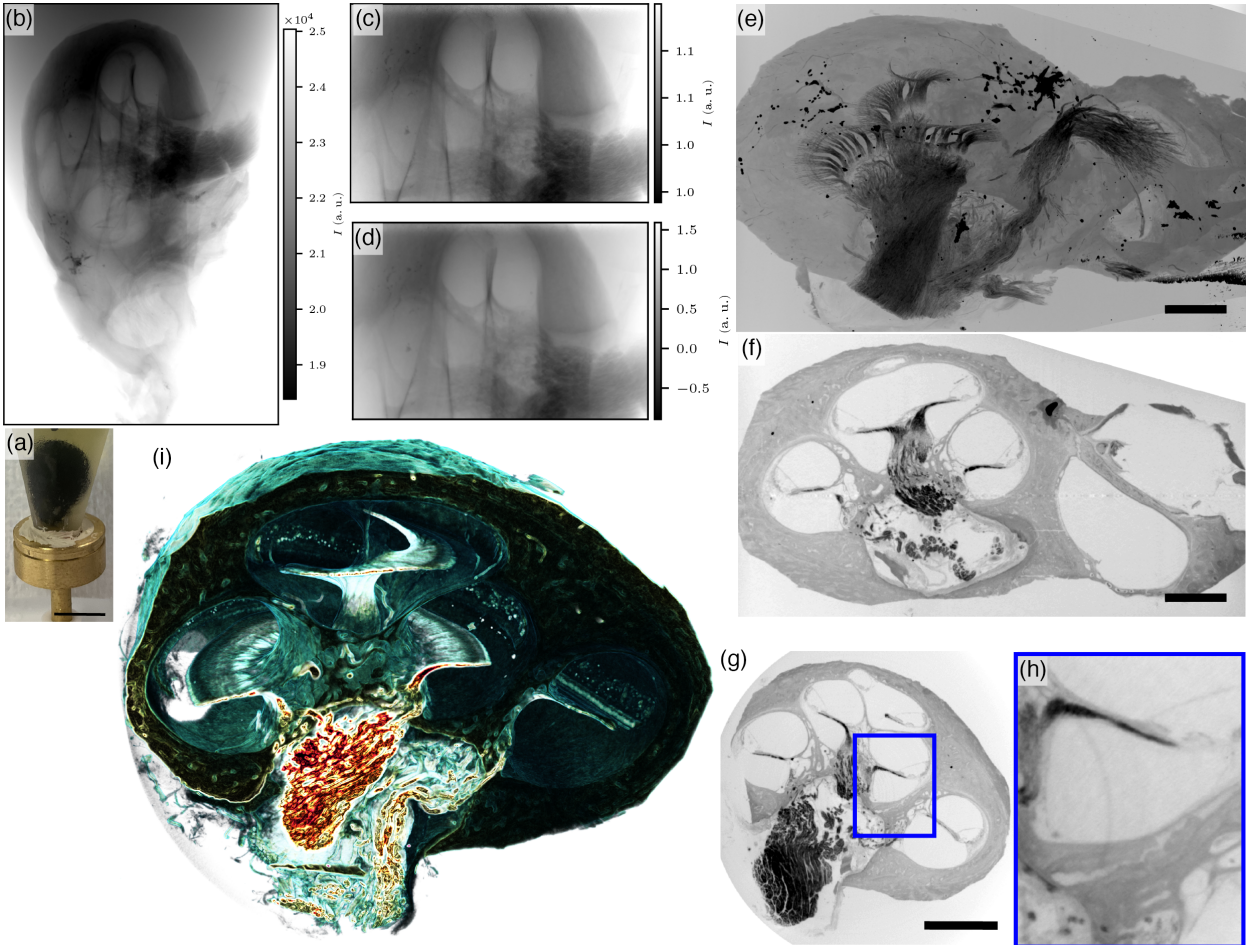
OTO-stained human cochlea scanned at the μ-CT-Setup. Note that (e)–(h) are rotated by 90 deg clockwise around the z axis with respect to (a)–(d). (a) Foto of mounted sample, scale bar 1 cm. (b) Example projection from an overview scan. (c) Example projection from ROI tomography. (d) BAC phase reconstruction of (c) with α=0.2 and γ=0.8. (e) MIP in the xz-direction, scale bar 2 mm. (f) Virtual xz-slice, scale bar 2 mm. (g) Virtual slice in the xy-direction. (h) Zoom into (g). (i) Volume rendering (gradient-based).

Finally, we extended our study to a first example of a human cochlea scanned in-house by the instrument described above to highlight that the imaging modality described here can be upscaled from a small animal to the much larger human cochlea. Again, the cochlea was OTO stained and stored in PBS for the measurements. Here we performed an overview scan with the microfocus source and the flatpanel detector in an upright position at a voxel size of 7.2  μm. Subsequently, we recorded an ROI zoom-in with the nanofocus source and the CCD camera at a voxel size of 3.0  μm (see Fig. 6).

## 4 Discussion and Outlook

The results show that state-of-the art μ-CT data recorded at in-house sources yield unprecedented image quality of small animal cochleae if they are stained by optimized protocols, such as the OTO stain, which is especially well suited for investigating nervous tissue. For overview scans of the whole organ with voxel sizes in the range of a few micrometers, it is sufficient to carefully select the acceleration voltage, source, and detector configuration. Higher resolution scans benefit from phase-contrast correction schemes of the projections applied before the tomographic reconstruction. Alternatively, the phase retrieval could also be carried out after tomographic reconstruction using the 3D dataset.^44^ The latter approach, which is based on the 3D Fourier transformation of the entire volume, offers the advantage of identifying the parameters more quickly.

Importantly, when stained suitably, specimens embedded in aqueous buffer can be investigated, which otherwise is not possible under in-house imaging conditions. Monochromatic and highly coherent SR, on the other hand, enables much higher sensitivity to small density contrasts in the tissue, which can be decoded by more rigorous phase retrieval schemes. These phase retrieval approaches are still based on approximations, such as an optically weak and chemically homogenous sample, at least when the common CTF scheme is used, but the image acquisition regime meets at least the idealized conditions of monochromaticity and lateral coherence. Typically, the FOV of undulator radiation is limited to a few mm due to the small beam divergence, so whole organ cochlea scans can only be implemented by stitching dozens of tomograms. Given the small scan time of 1 to 2 min of a single scan, this is perfectly feasible in terms of total acquisition time. The data presented here show that this is also possible at a high image quality and without radiation damage if parameters are suitably chosen, in particular the photon energy E. By this approach, the entire organ can be mapped at the sub-μm voxel size. We believe that these multi-scale approaches, which cover length scales from micro-anatomy to the histological level, will become extremely useful in future work, not only for cochlea imaging but similarly for other functional tissue.

The in-house μ-CT approach enabled by optimized staining protocols is also highly suitable for biomedical studies investigating special pathologies such as tissue degeneration associated with hearing disorders. Finally, we showed that the translation from small animal to human cochlea is also possible at surprising image quality already at a commercial μ-CT, opening up new opportunities for clinical pathology. In the future, we will extend the XPCT of the human cochlea also to SR, with the goal of achieving unprecedented 3D data at histological resolution. Finally, we note that the multi-scale approach can be extended to the nanometer range by cone beam recordings with nanofocused SR, as we have already shown for other tissues.^13^^,^^30^^,^^45^

## Data Availability

The 3D volume datasets obtained by XPCT presented in this paper are publicly available in GRO.data in Ref. 47.
